# Age-related changes in size, bone microarchitecture and volumetric bone mineral density of the mandible in the harbor seal (*Phoca vitulina*)

**DOI:** 10.1371/journal.pone.0224480

**Published:** 2019-10-24

**Authors:** Patricia Kahle, Tim Rolvien, Horst Kierdorf, Anna Roos, Ursula Siebert, Uwe Kierdorf

**Affiliations:** 1 Department of Biology, University of Hildesheim, Hildesheim, Germany; 2 Department of Osteology and Biomechanics, University Medical Center Hamburg-Eppendorf, Hamburg, Germany; 3 Department of Orthopedics, University Medical Center Hamburg-Eppendorf, Hamburg, Germany; 4 Department of Contaminant Research, Swedish Museum of Natural History, Stockholm, Sweden; 5 Institute of Terrestrial and Aquatic Wildlife Research, University of Veterinary Medicine Hannover, Hannover, Germany; Nanjing Medical University, CHINA

## Abstract

Detailed knowledge of age-related changes in the structure and mineralization of bones is important for interpreting osseous changes in wild mammals caused by exposure to environmental contaminants. This study analyzed mandibular size, microarchitecture and volumetric bone mineral density (vBMD) in harbor seals (n = 93, age range 0.5 months to 25 years) from the German North Sea. Bone microarchitecture and vBMD were assessed using high-resolution peripheral quantitative computed tomography (HR-pQCT). Significant differences were observed between the analyzed age classes (i) young juveniles (0.5–10 months), (ii) yearlings (12–23 months), and (iii) adults (12–25 years) for several of the variables, indicating an overall increase in cortical and trabecular area, cortical thickness and total and cortical vBMD with age. Furthermore, for juvenile animals (≤ 23 months), significant positive correlations with age were observed for mandible length and perimeter, cortical area, cortical thickness, trabecular separation, and total and cortical vBMD. The findings demonstrate a rapid increase in overall size, cortical dimensions and the degree of mineralization of the harbor seal mandible during the first two years after birth. Negative correlations with age existed for trabecular number and thickness as well as for trabecular bone volume fraction in the juveniles. The findings suggest a reduction in trabecular bone volume fraction with age, due to the bone trabeculae becoming thinner, less numerous and more widely spaced. Given the strong age dependence of most analyzed parameters, it is recommended to standardize samples with respect to age in future studies comparing microarchitecture and mineralization of harbor seal mandibles from different populations or different collection periods.

## Introduction

For several decades, both the German North Sea and the Baltic Sea have been exposed to high levels of contaminant input from industrial and agricultural sources, potentially leading to negative effects on marine mammal health [1–6]. These contaminants include persistent organic pollutants (POPs) that have a strong potential for biomagnification, resulting in a high contaminant load in top predators such as pinnipeds [7], cetaceans [8], and polar bears [9] from different regions. Due to their lipophilic character, POPs accumulate mainly in tissues with a high fat content.

Negative impacts of POPs and their metabolites on the immune, endocrine, and reproductive systems of different seal species have been repeatedly reported [7, 10–14]. In addition, a number of studies addressed the relationship between POP exposure and bone mineral density (BMD) in different species of Carnivora [15–22]. These studies were performed on dry skeletons from museum collections. Only in some cases, soft tissue samples from the studied animals were available to test for a direct link between BMD or bone structure and individual tissue levels of POPs. Where this was not the case, collection dates of the animals were used as a proxy for exposure levels to POPs, since the concentrations of these contaminants in the environment show marked temporal variation [15, 16, 21]. A positive association between ambient contaminant levels and 1) the prevalence of pathological skull changes and 2) the degree of fluctuating asymmetry in the skulls was indicated for harbor seals (*Phoca vitulina*) and grey seals (*Halichoerus grypus*) in different studies [23–25]. More recently, Pertoldi et al. [22] reported a higher BMD in non-pathological skulls of harbor seals compared to skulls showing pathological bone changes. The harbor seal is a top predator in the North Sea and the Baltic Sea [26, 27]. The species is common in the Wadden Sea, with an estimated population size of about 40,000 individuals in 2018 [28]. The harbor seal has a long life span and a rather small home range [29], thus making it a suitable bioindicator. Thus far, only one study on a marine mammal species, the grey seal, analyzed microstructural properties of the bones in addition to BMD [15].

It has been demonstrated that trabecular BMD and other bone properties vary markedly among different mammalian species used in experimental research [30]. This variation in baseline values of structural and compositional parameters of bone must be taken into account when comparing data from different species on the potential effects of POPs on bone. Another factor needing consideration in this context is ontogenetic variation in structural and compositional properties of bones within a species. This is particularly important when samples are compared that differ in their age composition, a situation not infrequently encountered in studies on wild mammals.

Age-related changes in bone microarchitecture and mineralization during pre- and postnatal development have previously been studied in mandibles of domestic pigs (*Sus scrofa domesticus*) [31, 32], but so far no data for seals are available. The principal aim of the present study was, therefore, to analyze the relationship between postnatal age and size (length, perimeter), bone microarchitecture, and volumetric BMD (vBMD) of mandibles in harbor seals from the German North Sea. In addition to changes in mandible size, we also expected to find changes in bone mineralization and structure with age in the animals.

As rapid skeletal growth occurs in the first years of life of harbor seals [33], a special focus of the investigation was put on juvenile animals. The method used in the present study, high-resolution peripheral quantitative computed tomography (HR-pQCT), provides both vBMD and bone microstructural data and has a higher resolution than previously used methods for measuring BMD in seals.

## Materials and methods

The study was performed on 93 clean (macerated and defatted) and dry left mandibles of Eastern Atlantic harbor seals (*Phoca vitulina vitulina*). Except for one adult individual originating from the island of Heligoland, all studied animals had been collected in the German part of the Wadden Sea. The analyzed mandibles were free from the pathological changes previously described in harbor seals (alveolar bone loss indicative of advanced periodontitis; alveolar bone exostosis, enlarged foramina) and hypothetically related to increased exposure to environmental pollutants [22, 24]. The studied specimens (S1 Table) are permanently deposited in the collection of the Zoological Institute of the University of Kiel (ZIK), Germany, and accessible upon request. Previous studies by our group on the harbor seal skull material from this collection addressed the spectrum and prevalence of dental anomalies and lesions [34] and of osteoarthritic changes of the temporomandibular joint [35]. Age determination on the seals had been performed by Abt [36], based on cement-layer-analysis in canines or gross morphological criteria for individuals younger than one year. Each mandible was labeled with a unique catalogue number, and information on sex, age, and date and locality of collection were given on a tag attached to the skull.

The analyzed mandibles originated from juveniles (age range: 0.5 to 23 months, mean age: 11 months, n = 66) and adult individuals (age range: 12 to 25 years, mean age: 14 years, n = 27). For statistical analysis, the juvenile subsample was further divided into the age classes “young juveniles” (age range: 0.5 to 10 months, mean age: 6 months; n = 25) and “yearlings” (age range: 12 to 23 months, mean age: 14 months; n = 41). Except for one individual (from the year 1961), all young juveniles had been collected in the period 1989 to 1994, while all adults had been collected between 1988 and 1994. Of the yearlings, 22 had been collected between 1974 and 1984, and 19 between 1988 and 1993.

Harbor seals reach sexual maturity at an age of about 3 to 6 years (females: 3 to 4 years, males: 4 to 6 years) [37]. To exclude sex-related differences in BMD, the adult subsample was composed only of mandibles obtained from male individuals. The juvenile subsample (age classes young juveniles and yearlings) included mandibles from females and males. These seals were sexually immature, and therefore no effect of sex on the analyzed variables was expected.

Mandibular length (from the infradentale to the posterior border of the condylar process) was measured with a caliper. Mandibular perimeter, microarchitecture and vBMD were assessed using high-resolution peripheral quantitative computed tomography (HR-pQCT, Xtreme-CT^®^, Scanco Medical, Bruettisellen, Switzerland) at 60kV, 900 μA, 100 ms integration time per rotation, and 82 μm voxel size. The scanner was calibrated daily by measuring a standardized hydroxyapatite (HA) phantom.

**Fig 1:**
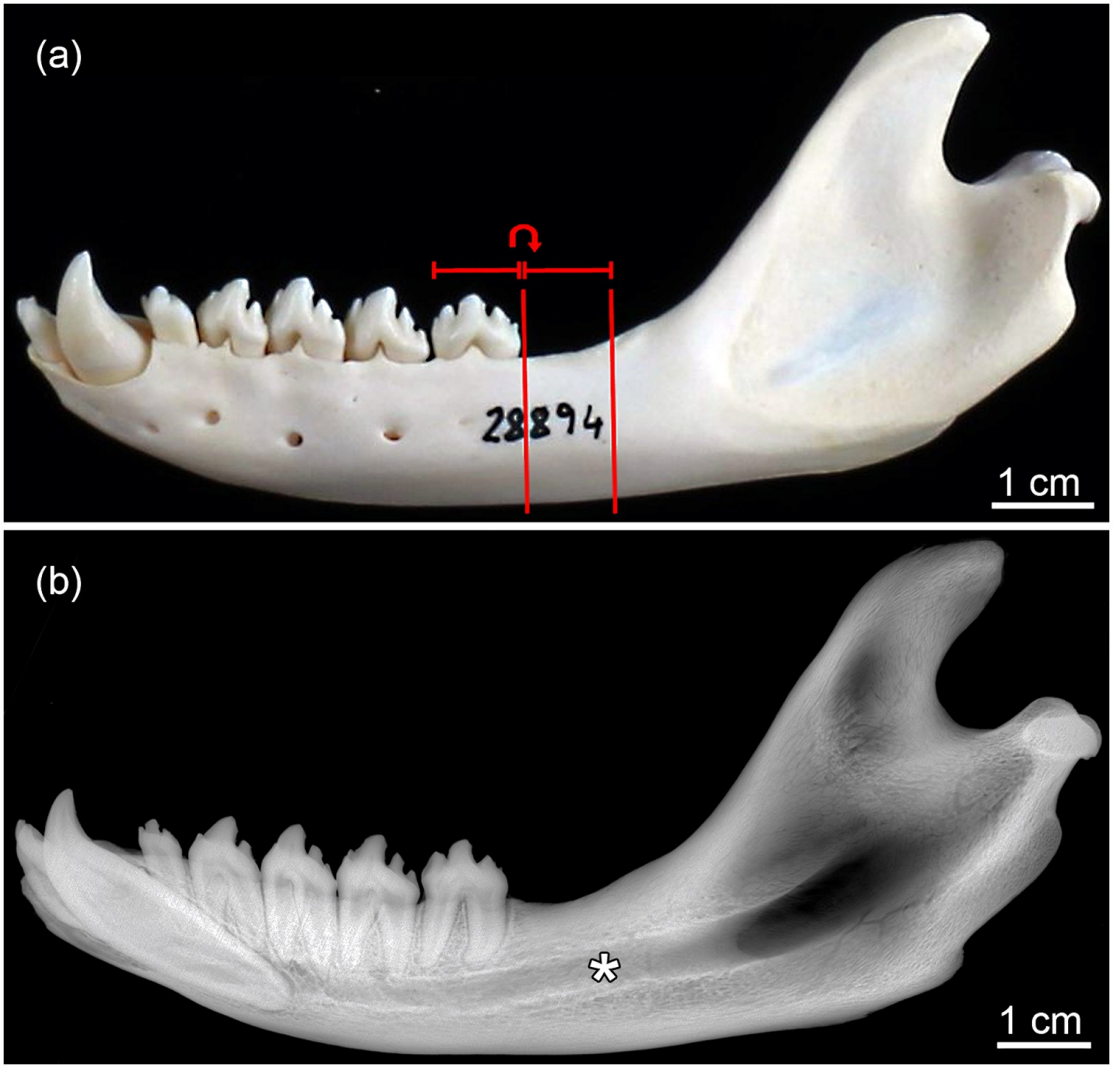
Mandibles of harbor seals (*Phoca vitulina*). (a) Lateral view of the left mandible of a 10-month-old individual. The measurement area in the retromolar region is indicated by the vertical bars. The width of the measured area equals the mesiodistal length of the tooth crown of the M_1_, the posteriormost of the five postcanine teeth. (b) X-ray image (lateromedial projection) of the left mandible of a 20-month-old individual, showing the distribution of compact and cancellous bone and the size of the mandibular canal (asterisk).

**Fig 2:**
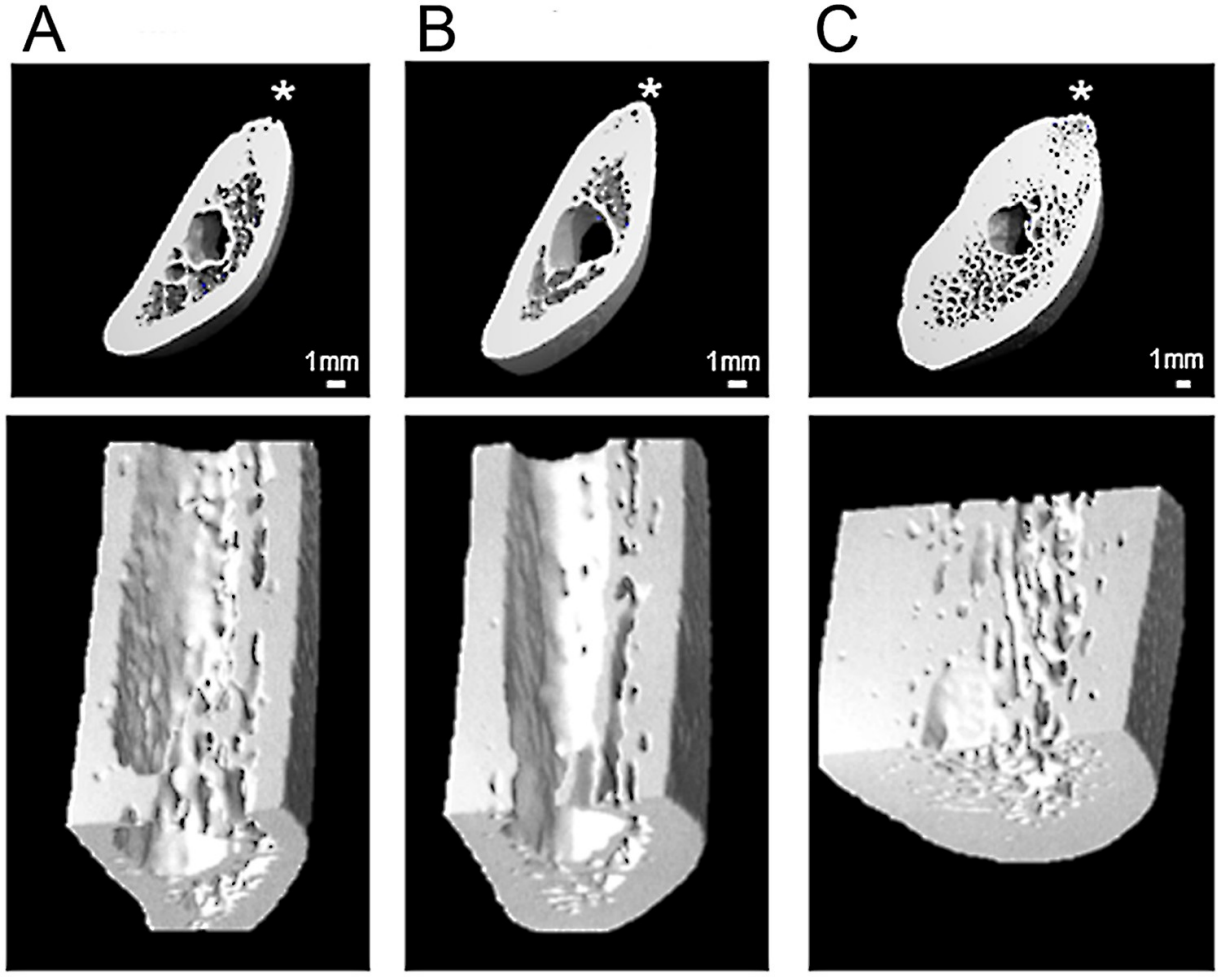
Virtual transverse sections of the retromolar region of the corpus of three left mandibles of harbor seals (*Phoca vitulina*) based on 3D-reconstructions using HR-pQCT data. Upper row: overviews, asterisks indicate dorsal borders of the mandibles; lower row: higher magnification of ventral portion of the mandibular corpus. A: young juvenile; B: yearling; C: adult individual. Note increase in cortical thickness with age.

Cross-sectional 3D-scans were performed (in air) in the retromolar area of the mandibular corpus, i.e., in the area between the M_1_ and the ascending mandibular ramus. As the amount and architecture of trabecular bone varies between different portions of the mandible [31, 38], we standardized the location of the scans and hence the volume of interest (VOI). For that, the mesiodistal length of the M_1_ crown or, when this tooth was missing due to postmortem loss, of the M_1_ tooth socket, was measured. Scanning was then performed in the area between the distal border of this tooth, or, in the case of its postmortem loss, the distal border of the M_1_ alveolus, and a point located one M_1_-tooth-length distal to this point (Fig 1). This region was chosen, because in their study on grey seal mandibles Lind et al. [15] also scanned the retromolar area. The VOI included the empty space of the mandibular canal (Figs 1 and 2).

Raw data were analyzed with an evaluation program (Scanco Medical). The following geometric, microarchitectural and mineralization parameters were obtained: bone perimeter (mm), cortical area (Ct.Ar, mm^2^), trabecular area (Tb.Ar, mm^2^), cortical thickness (Ct.Th; mm), trabecular thickness (Tb.Th; mm), trabecular number (Tb.N; 1/mm), trabecular separation (Tb.Sp; mm), ratio of trabecular bone volume to total volume [= trabecular bone volume fraction] (BV/TV; %), vBMD for the entire VOI = total vBMD (Tt.vBMD; mg HA/cm^3^), and vBMD for the cortical region (Ct.vBMD; mg HA/cm^3^). A fixed global threshold of 400 mg HA/cm^3^ was automatically applied by the program to segment cortical and trabecular bone. After visual inspection of the segmentation contours, modification by manual contouring was performed when necessary. Nomenclature and definition of the architectural variables follow the guidelines of Bouxsein et al. [39].

As some of the data in one or more of the three age classes (young juveniles, yearlings, adults) deviated from normality, variation among them was analyzed by the non-parametric Kruskal-Wallis test, followed by multiple comparison of mean ranks among the groups [40]. The relationship between age at death and each of the outcome variables in juvenile animals (≤ 23 months, n = 66) was studied by linear regression analysis and calculation of Pearson correlation coefficients. We further computed partial correlations, controlling for age, between Ct.Th and the trabecular parameters Tb.Th, and BV/TV in juvenile (n = 66) and adult animals (n = 27). In all statistical tests, p-levels < 0.05 were considered to indicate significance. The tests were performed with the program Statistica v. 13.3 (Tipco Software Inc.). Coefficients of variation were calculated to compare the variability in bone microarchitecture and bone mineral density between the grey seal mandibles analyzed by Lind et al. [15] and the harbor seal mandibles studied by us.

## Results

Data for the three analyzed age classes and the statistical evaluation of the differences among them are given in Table 1. Mandibular length and perimeter significantly increased from young juveniles over yearlings to adults. A corresponding significant increase from young juveniles to yearlings and further to adults was also observed for Ct.Ar and Ct.Th (Table 1, Fig 2). Tb.Ar, Tt.vBMD and Ct.vBMD were significantly higher in adults compared to the two younger age classes. Tb.Th was significantly higher in young juveniles than in yearlings, while Tb.Sp in the young juveniles was significantly lower than in the two older age classes. The relatively thick trabeculae of the young juveniles appeared plate-like compared to more strut-like trabeculae in the mandibles of the adult seals (Fig 2). BV/TV of young juveniles significantly exceeded that of yearlings and adults. Although for Tb.N the overall comparison among the three age classes (Kruskal-Wallis test) reached significance, none of the pairwise post-hoc comparisons was significant. No significant differences were observed for Tb.Th, Tb.N Tb.Sp, and BV/TV between yearlings and adults (Table 1).

**Table 1:** Data on size, bone microarchitecture and volumetric bone mineral density of the studied harbor seal (*Phoca vitulina*) mandibles, and statistical evaluation of the differences among age classes. Data are given as median (IQR), mean (SD), and Min.–Max. IQR = interquartile range, SD = standard deviation, ns = not significant.

Age class	Sample n		Length (cm)	Perimeter (mm)	Ct.Ar (mm^2^)	Ct.Th (mm)	Tb.Ar (mm^2^)	Tb.Th (mm)	Tb.N (1/mm)	Tb.Sp (mm)	BV/TV (%)	Tt.vBMD (mg HA/cm^3^)	Ct.vBMD (mg HA/cm^3^)
**(A) Young juveniles (0.5–10 months)**	25	Median (IQR)	10.4 (0.6)	37.9 (2.2)	32.0 (7.3)	0.85 (0.15)	24.7 (5.3)	0.154 (0.026)	1.09 (0.15)	0.769 (0.130)	15.5 (4.0)	576.4 (63.7)	813.2 (64.7)
		Mean (SD)	10.4 (0.4)	38.1 (1.6)	32.3 (5.3)	0.85 (0.12)	23.7 (3.9)	0.149 (0.021)	1.05 (0.11)	0.813 (0.109)	15.6 (2.6)	567.4 (51.3)	809.5 (47.3)
		Min.–Max.	9.6–11.2	35.5–41.8	22.4–41.6	0.59–1.06	16.2–30.8	0.095–0.183	0.81–1.23	0.656–1.093	11.0–19.8	461.0–638.5	722.5–878.5
**(B) Yearlings (12–23 months)**	41	Median (IQR)	11.2 (0.3)	41.1 (2.7)	45.2 (9.6)	1.06 (0.20)	26.7 (5.5)	0.133 (0.037)	1.00 (0.18)	0.889 (0.159)	13.0 (3.8)	611.2 (88.7)	863.2 (74.5)
		Mean (SD)	11.2 (0.3)	41.1 (2.2)	43.3 (8.2)	1.05 (0.17)	26.3 (4.9)	0.131 (0.029)	0.98 (0.14)	0.920 (0.170)	12.6 (3.0)	605.1 (73.0)	851.3 (54.7)
		Min.–Max.	10.7–12.2	35.9–46.9	22.4–64.9	0.62–1.43	13.4–35.8	0.059–0.187	0.67–1.20	0.702–1.384	6.7–19.3	452.8–781.5	728.8–943.7
**(C) Adults (12–25 years)**	27	Median (IQR)	14.4 (0.4)	57.8 (3.2)	131.1 (20.9)	2.25 (0.37)	52.5 (16.2)	0.142 (0.054)	0.98 (0.12)	0.893 (0.129)	13.0 (6.3)	759.8 (73.7)	1009.0 (44.1)
		Mean (SD)	14.4 (0.4)	57.7 (2.6)	127.4 (16.4)	2.20 (0.23)	53.8 (13.6)	0.134 (0.038)	0.98 (0.12)	0.904 (0.110)	12.9 (3.2)	756.0 (60.4)	997.9 (29.1)
		Min.–Max.	13.3–15.0	52.8–64.9	97.2–165.2	1.78–2.68	30.6–95.3	0.052–0.217	0.73–1.27	0.695–1.197	5.2–17.7	622.5–855.2	919.9–1032.8
**Kruskal-Wallis test**			H = 76.332, p < 0.0001	H = 69.383, p < 0.0001	H = 69.707, p < 0.0001	H = 67.908, p < 0.0001	H = 58.030, p < 0.0001	H = 6.335, p = 0.042	H = 6.153, p = 0.046	H = 9.889, p = 0.0071	H = 14.717, p = 0.0006	H = 51.998, p < 0.0001	H = 60.787, p < 0.0001
**Multiple comparisons**	(A) vs (B)		p < 0.0001	p = 0.0012	p = 0.0010	p = 0.0027	ns	p = 0.038	ns	p = 0.0195	p = 0.0008	ns	ns
	(A) vs (C)		p < 0.0001	p < 0.0001	p < 0.0001	p < 0.0001	p < 0.0001	ns	ns	p = 0.0135	p = 0.0062	p < 0.0001	p < 0.0001
	(B) vs (C)		p < 0.0001	p < 0.0001	p < 0.0001	p < 0.0001	p < 0.0001	ns	ns	ns	ns	p < 0.0001	p < 0.0001

**Fig 3:**
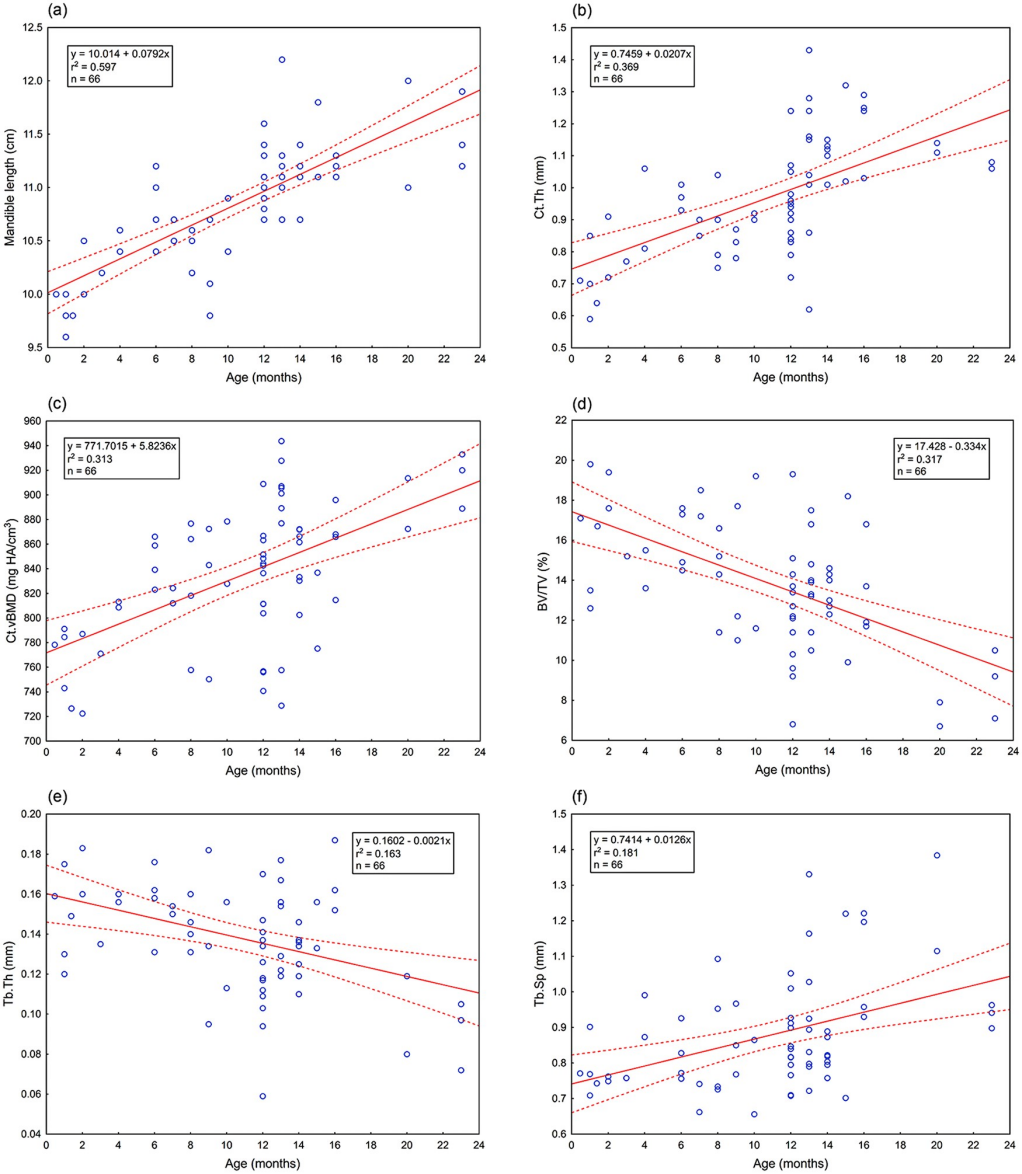
Relationship between age and morphological (length, cortical thickness), mineralization (cortical volumetric bone mineral density) and microarchitectural parameters (trabecular bone volume fraction, trabecular thickness and trabecular separation) in mandibles of juvenile harbor seal (*Phoca vitulina*) individuals (age range: 0.5 to 23 months, n = 66). The dashed lines indicate 95% confidence bands for the regressions.

In the juvenile animals (n = 66), significant positive correlations with age were observed for mandible length and perimeter, Ct.Ar, Ct.Th, Tb.Sp, Tt.vBMD, and Ct.vBMD (Table 2, Fig 3). The findings demonstrate a rapid increase in overall size, cortical dimensions and degree of mineralization of the harbor seal mandible during the first two years after birth. Negative correlations with age existed for Tb.Th, Tb.N, and BV/TV (Table 2, Fig 3). For the microarchitecture of the trabecular compartment of the mandibles this means that bone volume fraction was reduced with increasing age, due to the bone trabeculae becoming thinner, less numerous and more widely spaced.

**Table 2:** Results of correlation analysis (Pearson’s product-moment correlation coefficient and its significance) between age (range 0.5 to 23 months) and size, microarchitectural and bone mineral density variables of the mandibles of juvenile harbor seals, *Phoca vitulina* (age range: 0.5 to 23 months; n = 66); ns = not significant.

Variable	Correlation coefficient (r)	p-value
Length	0.773	< 0.0001
Perimeter	0.622	< 0.0001
Ct.Ar	0.648	< 0.0001
Ct.Th	0.608	< 0.0001
Tb.Ar	0.224	ns
Tb.Th	-0.404	0.0008
Tb.N	-0.350	0.004
Tb.Sp	0.426	0.0004
BV/TV	-0.563	< 0.0001
Tt.vBMD	0.391	0.001
Ct.vBMD	0.559	< 0.0001

Given are the nominal p-values. All significant correlations remained significant (adjusted p-value < 0.05) following Bonferroni correction to account for the increased risk of a type 1 error when performing multiple (n = 11) tests.

Partial correlations, controlling for age, between Ct.Th and the variables Tb.Th and BV/TV are given in Table 3. In the juvenile seals, significant partial correlations with Ct.Th existed for Tb.Th, while in adult seals this was the case for BV/TV and Tb.Th.

**Table 3:** Partial correlations, controlling for age, between cortical thickness and the variables Tb.Th and BV/TV in juvenile (age range: 0.5 to 23 months) and adult (age range: 12 to 25 years) harbor seals, *Phoca vitulina*; ns = not significant.

	Juvenile seals (n = 66)	Adult seals (n = 27)
Tb.Th	0.505, p < 0.0001	0.665, p = 0.0002
BV/TV	0.131, ns	0.526, p = 0.0058

## Discussion

While data on BMD are available for different marine mammals, with emphasis on the potential impact of environmental pollutants, the effect of age on the degree of bone mineralization has thus far been studied in greater detail only in polar bears [16–18, 20, 21]. Age-dependent variation of microarchitectural parameters in skeletal elements of marine mammals has not previously been addressed. The present study, for the first time, provides quantitative data on age-related changes of bone microarchitectural and mineralization parameters in harbor seal mandibles, with special emphasis on juvenile individuals.

Pronounced differences between age classes were found for all studied parameters except Tb.N. In addition, for juvenile seals, significant age-dependent increases of mandible length and perimeter, Ct.Ar, Ct.Th, Tb.Sp, Tt.vBMD, and Ct.vBMD were recorded. The changes in length, perimeter, Ct.Ar, Ct.Th, and Tb.Ar of the harbor seal mandibles reflect the increase in mandibular size and cortical robustness with age. The increase in vBMD with age observed in the juvenile individuals and the recorded differences among the three age classes (young juveniles, yearlings, adults) indicates that the degree of mineralization in the harbor seal mandibles increases over an extended period until the final bone mineral content is reached. This is regarded to reflect the process of secondary bone mineralization and the resulting gradual maturation of the bone mineral, which includes an increase in the number and size of the HA crystals as well as their crystallographic perfection [41, 42]. For ewes (*Ovis aries*) it has been reported that secondary mineralization in bone structural units is completed after 30 months [41]. An increase in mineralization of cancellous bone from about 500 to 850 mg HA/cm^3^ was recorded for the mandibular condyle of domestic pigs over the period from 8 weeks before birth to week 108 of postnatal age [32].

The mean Ct.Th reported for adult grey seals by Lind et al. [15], who used pQCT measurements, about double the value obtained by us for adult harbor seals (Table 4). Furthermore, higher Ct.Ar and lower Tb.Ar values compared to adult harbor seals are reported for the adult grey seals [15]. This results in a markedly higher ratio of average cortical to average trabecular bone area in the adult grey seals (range: 10.15–11.57) compared to the adult harbor seals (2.37). It is presently unclear to what extent these differences reflect actual variation between the two seal species compared to variation caused by differences in data acquisition and analysis.

**Table 4:** Comparison of bone microarchitecture and bone mineral density of mandibles from grey seals, *Halichoerus grypus*, [15] and harbor seals, *Phoca vitulina* (this study). SD = standard deviation, CV = coefficient of variation (%).

Study	Species, collection period	Sample n	Mean age (Min.–Max.)	Ct.Ar (mm^2^): mean (SD), CV	Tb.Ar (mm^2^): mean (SD), CV	Ct.Th (mm): mean (SD), CV	Ct.vBMD (mg HA/cm^3^): mean (SD), CV
Lind et al. [15]^1^	Grey seal, 1850–1955	9	15 years (9–23 years)	181.7 (32.4), 17.8	17.9 (4.5), 25.1	4.3 (0.6), 14.0	1174.8 (24.6), 2.1
	Grey seal, 1965–1985	22	11 years (4–15 years)	164.2 (28.6), 17.4	14.4 (5.6), 38.9	4.3 (0.5), 11.6	1162.1 (25.8), 2.2
	Grey seal, 1986–1997	12	10 years (4–15 years)	172.4 (52.0), 30.2	14.9 (5.2), 34.9	4.4 (1.0), 22.7	1135.5 (28.4), 2.5
This study	Harbor seal, 1988–1994)	27	14 years (12–25 years)	127.4 (16.4), 12.9	53.8 (13.6), 25.3	2.2 (0.2), 10.6	997.9 (29.1), 2.9
	Harbor seal, 1961–1994	66	11 months (0.5–23 months)	39.2 (9.0), 23.1	25.3 (4.7), 18.4	1.0 (0.2), 18.7	835.5 (55.5), 6.6

^1^Values given in Lind et al. [15] are means and standard errors of the mean. Standard deviations and coefficients of variation were calculated from these data.

The values recorded for Ct.vBMD in the adult harbor seals (mean: 997.9 mg HA/cm^3^) are lower than those (range of means: 1135.5–1174.8 mg HA/cm^3^) previously reported for mandibles of adult grey seals by Lind et al. [15] (Table 4). The latter values are similar to those obtained by pQCT analysis for Ct.vBMD (mean ± SE: 1153.5 ± 12.1 to 1185.2 ± 11.0 mg HA/cm^3^) of mandibles in a sham-treated group of domestic rabbits (*Oryctolagus cuniculus*) [43]. Using quantitative microradiography, a value of 1220 ± 140 mg HA/cm^3^ (mean ± SD) was reported for cortical bone of ewes after 30 months of mineralization [41].

The lower values for Ct.vBMD in the mandibles of the adult harbor seals compared to those of the adult grey seals [15], might be indicative of a higher remodeling activity in the former. This would lead to a higher volume fraction of younger, less mineralized bone tissue in the harbor seal mandibles. However, as the two studies did not use the same methods, the differences in mandibular Ct.vBMD between harbor and grey seals must be interpreted with caution. Further studies with standardized methods addressing possible interspecific variation of bone mineralization in seals are encouraged.

Regarding age-related changes in trabecular microarchitecture of the mandibles, for juvenile harbor seals our data indicate a decline in trabecular bone volume fraction due to a reduction in the number and thickness of the trabeculae and an increase in trabecular separation. Thus, with increasing age the trabecular system in the juveniles gets more delicate and widely spaced. No significant further change in trabecular microarchitecture occurred between yearlings and adult seals.

In their study on pig mandibles, Willems et al. [32] likewise observed a decrease in trabecular number and an increase in trabecular spacing from the late fetal stage to the adult individual. However, contrary to our findings in the harbor seals, trabecular thickness in the pig mandibles increased with age and, in consequence, age and trabecular bone volume fraction were positively correlated. This corresponds to the situation in humans where trabecular bone volume fraction also increases during the growth phase via a thickening of trabeculae until skeletal maturity is reached, while the number of trabeculae decreases [44, 45]. The reason for the discrepancy between harbor seals and other mammals in age-related changes of trabecular microarchitecture is currently unexplained, and future studies addressing age-related changes in trabecular microarchitecture of mandibles and other skeletal elements of seals are therefore required.

To our knowledge, the only previous study reporting data on bone mineralization in harbor seals is that by Pertoldi et al. [22]. These authors obtained CT scans of 34 skulls of adult harbor seals (24 males, 10 females) from three different locations (Wadden Sea, Limfjord, Kattegat) and used Hounsfield Units (HU) as a measure of the degree of mineralization. They found that non-pathological skulls had a significantly higher pooled HU number (denoting a higher vBMD) than skulls showing pathological changes in the form of alveolar exostosis, alveolar bone loss indicative of advanced periodontitis, and enlarged foramina. A direct comparison with the values obtained in our study is not possible, since absolute values for vBMD (mg HA/cm^3^) are not reported by Pertoldi et al. [22]. However, we wish to emphasize that none of the skulls analyzed by us exhibited the pathological changes listed above.

So far, HR-pQCT imaging has only rarely been used to study bones of marine mammals [46, 47]. A number of previous studies [16–18, 20,21] used dual-energy X-ray absorptiometry (DXA), a method providing aBMD data [48]. This method does not allow for a separate assessment of the cortical and trabecular compartment or for obtaining data on bone microarchitecture. The present study, however, has highlighted the importance of a separate investigation of both types of bone to characterize the changes in the different bone compartments.

In their grey seal study, Lind et al. [15] found that trabecular vBMD of the radius was significantly higher in specimens from a period with decreasing ambient levels of POPs (1986–1997, “low-pollution-period”) compared to animals collected during the period of highest release of POPs into the environment (1965–1985, “high-pollution-period”). Measurements of cortical vBMD in mandibles revealed significantly higher values in animals from both, the period prior to the onset of POP-production (1850–1955, “pre-pollution-period”) and the high-pollution period compared to the low pollution period. The differences between sampling periods remained significant when animal age was taken into account as a confounding variable.

Several studies on the relationship between BMD and exposure to POPs were conducted in the polar bear (*Ursus maritimus*) [16–18, 20, 21]. Sonne et al. [16, 17] reported a negative relationship between areal BMD (aBMD) of skulls and tissue levels of some contaminants. Values of subadult and adult males were higher in specimens collected in the period 1892–1932 than in those from the period 1966–2002, and in those with low POP levels in adipose tissue compared to those with high levels. For bacula, a (non-signifcant) trend of decreasing aBMD with increasing PCB concentrations was established for the eight analyzed polar bear subpopulations [18]. Conflicting findings were more recently reported by Daugaard-Petersen et al. [20, 21], who found a positive correlation between aBMD of skulls and bacula and the body load of several contaminants in polar bears. However, analyses of skulls showed a decline in aBMD from 1892 to 2015 in adult males from the East Greenland population. Polar bears collected in the period 1892–1936 had significantly higher aBMD than those from the period 1966–2015. In contrast, no significant relationship between contaminant load and aBMD were found in polar bears from Svalbard. The inconsistencies in the findings of the above studies might be partly explained by a sampling bias, as both Sonne et al. [16–18] and Daugaard-Petersen et al. [20, 21] reported a dependency of aBMD on age in polar bears. These findings underscore the necessity of taking age-related variation of BMD into account when addressing the potential effects of environmental pollutants on bone mineralization.

It has been shown that the accuracy of HR-pQCT measurement of trabecular vBMD is influenced by variation in cortical thickness [49]. Overestimation of trabecular vBMD increased proportional to cortical thickness due to beam hardening artifacts, which were not entirely eliminated by the hardening correction algorithms used by the manufacturer’s software. It was further shown that the error in trabecular vBMD propagated to other parameters, particularly Tb.Th and BV/TV, that are derived from the density of the trabecular compartment [49]. Large errors occurred in the case of co-occurrence of high Ct.Th and low trabecular vBMD, a combination that is not uncommon in healthy human individuals [49]. Our finding of significant partial correlations (controlling for age) between Ct.Th and Tb.Th in both juvenile and adult harbor seals as well as between Ct.Th and BV/TV in adults could indicate such an effect of Ct.Th on trabecular parameters, although no definite conclusion can be drawn on the basis of the available data.

More recently, correction values for HR-pQCT-derived trabecular measurements have been published for the human tibia and radius [50]. When such correction values are lacking, as in the case of the seal mandibles, it should be attempted to minimize the error caused by variation in cortical thickness. Thus, when comparing data from different populations of harbor seals or different collection periods within a population, it is recommended to standardize the samples with respect to age, as cortical thickness has been shown to be a strongly age-dependent parameter especially in younger seals. With respect to the present study, the observed effect of cortical thickness on trabecular parameters indicates the need for a cautious interpretation of the results.

In conclusion, the present study, for the first time, reports basic data on age-related variation of bone microarchitecture and volumetric mineral density in harbor seal mandibles using HR-pQCT. These data can be used in future studies assessing the effects of exposure to environmental pollutants on bone structure and bone mineralization in seals. It has further been demonstrated that the analysis of skeletal material from museum collections is a means of retrospectively obtaining information on the health status of mammal populations and its changes over time. Previous studies on skeletal material from harbor seals inhabiting the German North Sea analyzed the prevalence and spectrum of dental anomalies and lesions [34] and of osteoarthritic changes of the temporomandibular joint [35]. By combining the results of the present and our previous studies with those of future studies addressing other issues, e.g. alveolar bone pathology due to periodontitis, it will be possible to provide a comprehensive assessment of skeletal and dental health in the studied population.

## Supporting information

S1 Table Overview of the harbor seal specimens whose mandibles were analyzed in the present study. The specimens are permanently deposited in the collection of the Zoological Institute of the University of Kiel (ZIK), Germany, and accessible upon request.
(DOCX)
