# Supplementary material for: Age-related changes in size, bone microarchitecture and volumetric bone mineral density of the mandible in the harbor seal (Phoca vitulina)
Source: PLoS One. 2019 Oct 24;14(10):e0224480. doi: 10.1371/journal.pone.0224480 (PMC6812799; doi:10.1371/journal.pone.0224480)
Supplement: S1 Table — The specimens are permanently deposited in the collection of the Zoological Institute of the University of Kiel (ZIK), Germany, and accessible upon request. (DOCX) [file pone.0224480.s001.docx]

**S1 Table**. Overview of the harbor seal specimens whose mandibles were analyzed in the present study. The specimens are permanently deposited in the collection of the Zoological Institute of the University of Kiel (ZIK), Germany, and accessible upon request.

| \| Specimen ID: ZIK \| \| --- \| | \| Sex \| \| --- \| | \| Collection year \| \| --- \| | \| Age at death (months) \| \| --- \| |
| --- | --- | --- | --- | --- | --- | --- | --- |
| 1966 | female | 1961 | 6 |
| 16721 | male | 1974 | 14 |
| 16725 | male | 1974 | 14 |
| 16739 | male | 1974 | 14 |
| 17102 | male | 1975 | 13 |
| 17124 | male | 1975 | 13 |
| 17163 | male | 1975 | 16 |
| 17236 | male | 1975 | 16 |
| 17239 | male | 1975 | 14 |
| 17509 | male | 1976 | 20 |
| 17627 | male | 1976 | 14 |
| 19326 | male | 1977 | 13 |
| 19328 | male | 1977 | 14 |
| 19330 | male | 1977 | 14 |
| 21980 | male | 1981 | 20 |
| 21985 | male | 1981 | 13 |
| 22047 | male | 1981 | 16 |
| 22097 | male | 1989 | 4 |
| 22100 | male | 1989 | 4 |
| 22824 | male | 1981 | 15 |
| 22862 | male | 1982 | 12 |
| 23130 | male | 1982 | 16 |
| 23970 | male | 1983 | 12 |
| 24885 | male | 1984 | 12 |
| 25715 | male | 1983 | 13 |
| 27672 | male | 1988 | 23 |
| 27761 | male | 1988 | 300 |
| 27774 | male | 1988 | 12 |
| 27785 | male | 1988 | 23 |
| 27833 | male | 1988 | 156 |
| 27842 | male | 1988 | 23 |
| 27852 | male | 1988 | 12 |
| 27857 | male | 1988 | 12 |
| 27866 | male | 1988 | 12 |
| 27918 | male | 1988 | 12 |
| 27921 | male | 1988 | 12 |
| 27932 | male | 1988 | 300 |
| 27993 | male | 1988 | 12 |
| 28036 | male | 1988 | 12 |
| 28056 | male | 1988 | 12 |
| 28193 | male | 1988 | 156 |
| 28347 | male | 1988 | 156 |
| 28362 | male | 1988 | 156 |
| 28434 | male | 1988 | 156 |
| 28473 | female | 1989 | 8 |
| 28678 | male | 1989 | 10 |
| 28735 | male | 1988 | 300 |
| 28894 | male | 1989 | 10 |
| 28992 | male | 1989 | 1 |
| 29107 | male | 1988 | 156 |
| 29142 | male | 1988 | 156 |
| 29165 | male | 1988 | 156 |
| 29215 | male | 1988 | 156 |
| 29297 | male | 1988 | 156 |
| 29334 | male | 1988 | 144 |
| 29375 | male | 1988 | 144 |
| 29404 | male | 1988 | 156 |
| 29518 | male | 1988 | 156 |
| 29543 | male | 1988 | 144 |
| 29819 | female | 1990 | 9 |
| 29820 | male | 1989 | 2 |
| 29971 | male | 1990 | 0.5 |
| 29972 | male | 1990 | 1 |
| 29973 | female | 1990 | 3 |
| 30030 | female | 1989 | 6 |
| 30221 | male | 1991 | 7 |
| 30275 | male | 1991 | 8 |
| 30480 | female | 1991 | 12 |
| 30482 | male | 1991 | 144 |
| 30500 | female | 1991 | 13 |
| 30605 | female | 1991 | 13 |
| 30606 | male | 1991 | 13 |
| 30637 | female | 1991 | 15 |
| 30779 | male | 1991 | 156 |
| 30951 | female | 1992 | 9 |
| 30952 | male | 1992 | 7 |
| 30953 | male | 1992 | 8 |
| 30996 | male | 1992 | 144 |
| 30998 | male | 1992 | 13 |
| 31016 | female | 1992 | 9 |
| 31017 | male | 1992 | 2 |
| 31041 | male | 1992 | 1 |
| 31196 | male | 1993 | 144 |
| 31215 | female | 1993 | 8 |
| 31247 | male | 1993 | 168 |
| 31274 | male | 1993 | 12 |
| 31452 | female | 1994 | 6 |
| 31453 | male | 1994 | 6 |
| 31455 | male | 1994 | 1 |
| 31458 | male | 1994 | 168 |
| 31524 | male | 1994 | 168 |
| 31555 | male | 1994 | 168 |
| 31558 | male | 1994 | 156 |
